# Calcifediol Treatment and Hospital Mortality Due to COVID-19: A Cohort Study

**DOI:** 10.3390/nu13061760

**Published:** 2021-05-21

**Authors:** Juan F. Alcala-Diaz, Laura Limia-Perez, Ricardo Gomez-Huelgas, Maria D. Martin-Escalante, Begoña Cortes-Rodriguez, Jose L. Zambrana-Garcia, Marta Entrenas-Castillo, Ana I. Perez-Caballero, Maria D. López-Carmona, Javier Garcia-Alegria, Aquiles Lozano Rodríguez-Mancheño, Maria del Sol Arenas-de Larriva, Luis M. Pérez-Belmonte, Irwin Jungreis, Roger Bouillon, Jose Manual Quesada-Gomez, Jose Lopez-Miranda

**Affiliations:** 1 Internal Medicine Department, IMIBIC/Reina Sofia University Hospital/University of Córdoba, Avda. Menéndez Pidal s/n, 14004 Córdoba, Spain; juanf.alcala.sspa@juntadeandalucia.es (J.F.A.-D.); laura_limia@hotmail.com (L.L.-P.); anabelperezcaballero@gmail.com (A.I.P.-C.); 2 CIBER Fisiopatologia Obesidad y Nutrición (CIBEROBN), Instituto de Salud Carlos III, 28029 Madrid, Spain; 3 Internal Medicine Department, Regional University Hospital of Málaga, Avenida de Carlos Haya, s/n, 29010 Málaga, Spain; ricardogomezhuelgas@hotmail.com (R.G.-H.); mdlcorreo@gmail.com (M.D.L.-C.); luismiguelpb1984@gmail.com (L.M.P.-B.); 4 Biomedical Research Institute of Málaga (IBIMA), University of Málaga (UMA), Avenida de Carlos Haya, s/n, 29010 Málaga, Spain; 5 Internal Medicine Department, Hospital Costa del Sol, Agencia Sanitaria Costa del Sol, 29603 Marbella, Málaga, Spain; mmartinescalante@gmail.com (M.D.M.-E.); jalegria@hcs.es (J.G.-A.); 6 Internal Medicine Department, Alto Guadalquivir Hospital, Andújar, 23740 Jaén, Spain; begocortesrod@gmail.com (B.C.-R.); aqlozanorod@gmail.com (A.L.R.-M.); 7 Internal Medicine Department, Hospital de Montilla, Agencia Sanitaria Alto Guadalquivir, 14550 Córdoba, Spain; jlzambrana@ephag.es; 8 Pneumology Department, Reina Sofia University Hopital. Avda, Menendez Pidal s/n, 14004 Córdoba, Spain; marenca@gmail.com (M.E.-C.); arlam23@hotmail.com (M.d.S.A.-d.L.); 9 Department of Medicine, University of Málaga (UMA), Red de Investigación en Servicios de Salud en Enfermedades Crónicas (REDISSEC), 29071 Málaga, Spain; 10 MIT Computer Science and Artificial Intelligence Laboratory, Cambridge, MA 02139, USA; ILJungr@csail.mit.edu; 11 Broad Institute of MIT and Harvard, Cambridge, MA 02142, USA; 12 Laboratory of Clinical and Experimental Endocrinology, Department of Chronic Diseases, Metabolism and Ageing, KU Leuven, Herestraat, ON 1/902, 3000 Leuven, Belgium; roger.bouillon@kuleuven.be; 13 IMIBIC. CIBER de Fragilidad y Envejecimiento Saludable, Hospital Universitario Reina Sofía, Universidad de Córdoba, Fundación Progreso y Salud, Avda. Menéndez Pidal s/n, 14004 Córdoba, Spain; md1qugoj@uco.es

**Keywords:** COVID-19, calcifediol, SARS-CoV-2, COVID-19 drug treatment, vitamin D

## Abstract

Context. Calcifediol has been proposed as a potential treatment for COVID-19 patients. Objective: To compare the administration or not of oral calcifediol on mortality risk of patients hospitalized because of COVID-19. Design: Retrospective, multicenter, open, non-randomized cohort study. Settings: Hospitalized care. Patients: Patients with laboratory-confirmed COVID-19 between 5 February and 5 May 2020 in five hospitals in the South of Spain. Intervention: Patients received calcifediol (25-hydroxyvitamin D_3_) treatment (0.266 mg/capsule, 2 capsules on entry and then one capsule on day 3, 7, 14, 21, and 28) or not. Main Outcome Measure: In-hospital mortality during the first 30 days after admission. Results: A total of 537 patients were hospitalized with COVID-19 (317 males (59%), median age, 70 years), and 79 (14.7%) received calcifediol treatment. Overall, in-hospital mortality during the first 30 days was 17.5%. The OR of death for patients receiving calcifediol (mortality rate of 5%) was 0.22 (95% CI, 0.08 to 0.61) compared to patients not receiving such treatment (mortality rate of 20%; *p* < 0.01). Patients who received calcifediol after admission were more likely than those not receiving treatment to have comorbidity and a lower rate of CURB-65 score for pneumonia severity ≥ 3 (one point for each of confusion, urea > 7 mmol/L, respiratory rate ≥ 30/min, systolic blood pressure < 90 mm Hg or diastolic blood pressure ≤ 60 mm Hg, and age ≥ 65 years), acute respiratory distress syndrome (moderate or severe), c-reactive protein, chronic kidney disease, and blood urea nitrogen. In a multivariable logistic regression model, adjusting for confounders, there were significant differences in mortality for patients receiving calcifediol compared with patients not receiving it (OR = 0.16 (95% CI 0.03 to 0.80). Conclusion: Among patients hospitalized with COVID-19, treatment with calcifediol, compared with those not receiving calcifediol, was significantly associated with lower in-hospital mortality during the first 30 days. The observational design and sample size may limit the interpretation of these findings.

## 1. Introduction

One of the most critical challenges facing contemporary medicine and public health systems in the world has emerged from the 2019 coronavirus (COVID-19) pandemic [1]. The severity of a SARS-CoV-2 infection can range from asymptomatic or mild respiratory symptoms to the development of respiratory failure, multiorgan failure, and death [2]. On 31 January 2020, the World Health Organization (WHO) announced that COVID-19 was labelled a Public Health Emergency of International Importance (PHEIC), and by 18 April 2021, it had affected over 141,334,774 confirmed cases with 3,024,317 deaths reported globally [3]. However, there are still many unclear issues related to transmission, infection, and treatment [1]. Since the beginning of the pandemic, intense pressure has been put on clinicians and researchers to provide advanced treatments to save lives. However, the pathophysiology of severe COVID-19 is very complex. It is a potentially lethal combination of immunopathogenic and immunoprotective responses in a prothrombotic environment [4]. Unfortunately, no single mechanism or pathway discovered so far explains the entire pathophysiology. The problem is that there were only a few successful therapies available, and still fewer have demonstrated efficacy compared to no treatment in clinical trials [1]. It has and still is challenging to treat patients with coronavirus 2019 (COVID-19). Specialists face distressing emergencies in the intensive care unit where, at the start of the pandemic, almost 20% of the hospitalized patients (with COVID-19) developed Acute Respiratory Distress Syndrome (ARDS) and, despite recent advances in mechanical ventilation and supporting treatment methods, about 65% of patients with ARDS died [5], explaining why 25% of critically ill patients with severe COVID-19 died in the first outbreak of the pandemic [6].

At the beginning of the pandemic, in the absence of specific COVID-19-causal treatments clearly effective on mortality, several approved or investigational drugs with in vitro activity against SARS-CoV-2 replication, including antiviral drugs such as lopinavir-ritonavir, remdesivir, hydroxychloroquine, anti-parasitic ivermectin, and different immunomodulatory medications, were proposed as potentially useful [7]. However, the WHO [8] has generated a strong recommendation against the use of hydroxychloroquine/chloroquine or lopinavir/ritonavir for treatment of COVID-19 of any severity, restricting the use of ivermectin only to clinical trials. For remdesivir, effective in reducing recovery time in adults hospitalized with COVID-19 [9], the WHO has established a conditional recommendation against administering remdesivir in addition to usual care; however, for dexamethasone or other corticosteroids, which are the only therapies that have been shown to reduce mortality so far in patients with severe disease requiring mechanical ventilation of high-flow oxygen [10], the WHO maintains a strong recommendation for the use of systemic corticosteroids for severe or critically ill COVID-19 patients, with a conditional recommendation against their use in patients with mild/moderate COVID-19. Recently, the FDA has approved by Emergency Use Authorization baricitinib in combination with remdesivir in patients requiring invasive mechanical ventilation or extracorporeal membrane oxygenation [11] as well as the neutralizing antibody bamlanivimab (LY-CoV555) [12].

Moreover, due to the absence of a specific treatment for ARDS, its management consists of general supportive care required for all critically ill hospitalized patients (prevention of blood clots, infection control, early nutritional assistance, and stress ulcer prophylaxis) as well as the use of ventilation and oxygen therapy [13,14]. In this respect, it has been suggested that the vitamin D endocrine system (VDES), by its extra-skeletal actions especially on the lung and the immune system, is a facilitator of immunocompetence with respect to both innate and adaptive immunity [13,15,16,17,18].

In this context, the stimulation of the vitamin D receptor (VDR) of the VDES has been proposed to reduce acute respiratory distress syndrome (ARDS), cardiac and coagulopathy risk, and possibly death rates in patients with COVID-19 [15,16,17,19,20]. Supported by this rationale, some clinical trials and studies of various types are currently underway to test the possible benefit of using oral cholecalciferol (vitamin D3) or calcifediol (also known as 25-hydroxyvitamin D₃) on patients with COVID-19 [17].

Cholecalciferol (or vitamin D3), obtained through cutaneous synthesis under UV-B light and in small amounts from the diet, is the threshold nutrient of the vitamin D endocrine system (VDES). Transported by vitamin D-binding protein (DBP), it is converted to 25-hydroxyvitamin D_3_ (25OHD) or calcifediol in the liver, primarily through the action of 25 hydroxylase. Levels of 25OHD are used by health authorities and scientific societies in America and Europe to establish the status of normality, the definition of vitamin D deficiency, and the degrees of insufficiency of the same, upon which to establish dietary reference intake values for vitamin D as well as the control of vitamin D deficiency, insufficiency, or excess in the population [21].

Calcifediol is a prohormone of VDES that serves as a substrate for the synthesis of 1,25(OH)2D or calcitriol via 1-hydroxylase (CYP2721B) in the kidney and multiple body cells. Calcitriol functions as a hormone and binds at nuclear level to the vitamin D receptor (VDR) with high affinity, controlling the expression of many genes with a wide range of functional activities [13].

By its relative potency and pharmacokinetic characteristics, when compared to cholecalciferol, oral calcifediol causes a faster increase in 25OHD serum levels [22,23].

The aim of our study was to compare the administration of oral calcifediol in conjunction with best available therapy versus best available therapy only and clinical outcomes among hospital in-patients diagnosed with COVID-19.

## 2. Materials and Methods

Calcifediol was approved by the Ethics Committee for the treatment of COVID-19 in the Reina Sofía University Hospital, Córdoba, Spain (EU) (Act-29/2020). Physicians formally informed patients or legal representatives about the calcifediol treatment and recorded their consent in the hospital’s electronic medical record. The retrospective data collection and analysis of the patients included in this study were approved by the Málaga provincial research ethics committee (protocol code “Registro-SEMI-COVID-19”, 27 March 2020).

### 2.1. Study Design, Sites, and Participants

**Figure 1:**
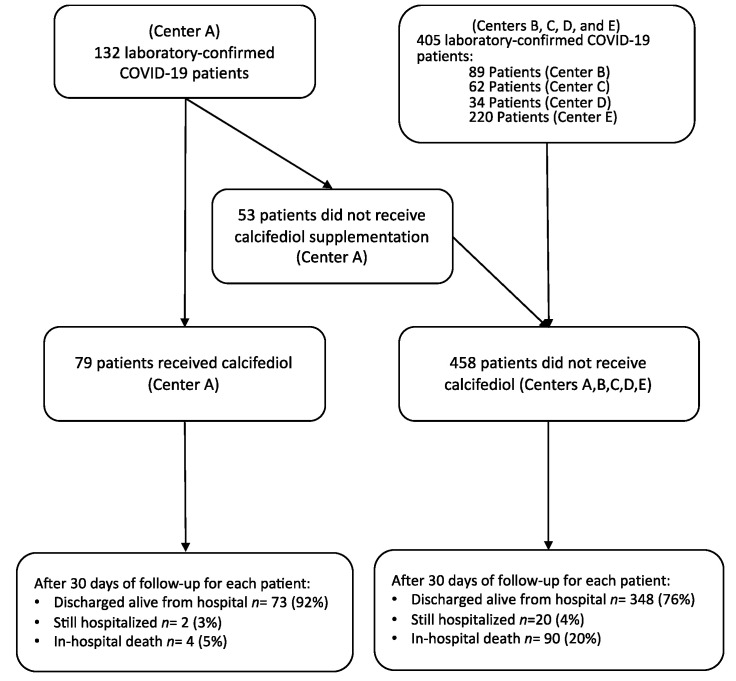
Flow chart of patients included in the study. Center A, Hospital Universitario Reina Sofia (Córdoba, Spain); Center B, Hospital Costa del Sol (Marbella, Spain); Center C, Hospital Alto Gualdalquivir (Andújar, Spain); Center D, Hospital Montilla (Córdoba, Spain); Center E, Hospital Universitario Regional (Málaga, Spain).

This retrospective, multicenter cohort study included 537 patients with a clinical picture of acute respiratory infection by COVID-19, radiographic signs of viral pneumonia, and positive SARS-CoV-2 polymerase chain reaction, admitted between 5 February and 5 May 2020 in 5 public health system hospitals in the South of Spain in the regions of Córdoba, Málaga, and Jaén (Hospital Universitario Reina Sofia, Córdoba; Hospital Universitario Regional, Málaga; Hospital Costa del Sol, Marbella; Hospital de Montilla; and Hospital Alto Guadalquivir, Andújar). The study included all sequentially hospitalized patients excluding 76 subjects already reported as part of the randomized pilot study previously reported of COVID-19 patients conducted in Hospital Universitario Reina Sofia [20]. The pharmacy and ethics committee authorized this hospital to use calcifediol treatment (as applied in the pilot trial) at the specialist’s discretion. No standardized criteria were used to indicate the use of calcifediol treatment. The different healthcare professionals involved in the treatment of the patients described in this study, composed of multidisciplinary teams, indicated at their discretion and under their clinical reasoning the use of the different therapeutic options used to address the infection. In the other hospitals, it was not included as it was not authorized by their respective committees. Medical records were analyzed for medications, pre-existing diseases, clinical measures on admission, outcomes, and adverse effects. Flow-chart of the study is shown in Figure 1.

### 2.2. Laboratory Analysis and Respiratory Function Test

Clinical specimens required for SARS-CoV-2 diagnostic procedures were obtained on admission by nasopharyngeal exudate sampling following WHO guidelines and recommendations. RNA extraction and real-time RT-PCR (rtRT-PCR) were performed at the local Central Microbiology Laboratory (Code 202 MagCore^®^ Viral Nucleic Acid Extraction Kit by Kapa Biosystems Inc. (Wilmington, MA, USA), Allplex™ 2019-nCoV Assay by Seegene Inc. (Seoul, Korea), or VIASURE SARS-CoV-2 real-time PCR Detection Kit by CerTest Biotec S.L. (Zaragoza, Spain)).

After blood sample collection, we determined a complete blood count using flow cytometry (ADVIA 2120i, Siemens Healthineers, Erlangen, Germany), coagulation study including d-dimer (coagulation and immunoturbidimetric assay on ACL TOP 700, Instrumentation Laboratory/Werfen), and others parameters related to renal and liver function, lactate dehydrogenase (spectrophotometric assay on Advia chemistry 2400 XPT, Siemens Healthineers, Erlangen, Germany), ferritin, C-reactive protein (CRP) (immunoturbidimetric assay on Advia chemistry 2400 XPT, Siemens Healthineers, Erlangen, Germany), and IL-6 (chemiluminescent immunoassay on Advia Centaur XPT, Siemens Healthineers, Erlangen, Germany). O2 saturation (SaO2) and chest radiography were determined in all patients on admission. An expert team of thoracic radiologists evaluated all radiographic evidence. The Berlin definition was used to define ARDS [24]. The CURB-65 score for severity of pneumonia (CURB-65) was calculated according to its international definition [25].

### 2.3. Procedures

According to hospital protocol, all hospitalized patients received the best available treatment for SARS-CoV-2 infection and standard care for pre-existing comorbidities. In addition, patients from the Hospital Universitario Reina Sofia (*n* = 132) were categorized into 2 treatment groups based on having received from admission: (1) oral calcifediol (25-hydroxyvitamin D_3_) in soft gelatin capsules (0.532 mg), then oral calcifediol (0.266 mg) on day 3 and 7, and then weekly until discharge or ICU admission following the posology of the pilot study from Hospital Universitario Reina Sofia, Córdoba [20] or (2) no treatment with calcifediol. All patients or legal representatives gave their informed consent before initiating treatment with calcifediol, according to the protocol approved by the Pharmacy Committee and by the Ethics Committee for the Treatment of COVID-19 of the Hospital Universitario Reina Sofía, Córdoba, Spain. Patients from the other four centers did not receive treatment with calcifediol, and they were included in the analysis as not-treated patients.

### 2.4. Outcome

We measured the rate of in-hospital deaths in the first 30 days after admission as the primary outcome. Our hypothesis in this retrospective study was that treatment with calcifediol would reduce the risk of death.

### 2.5. Statistical Analysis

To assess the association between treatment groups and clinical variables measured, χ2 test or Fisher’s exact test were used for categorical variables and Student’s *t*-test or Kruskal–Wallis test for continuous ones. By multivariable logistic regressions, we calculated the odds ratio of mortality risk by group of treatment, also adjusting by several potential confounders (date of hospitalization before or after approval of calcifediol treatment in Center A, age, gender, center, diabetes, chronic lung disease, smoking status, hypertension, coronary artery disease, cerebrovascular disease, congestive heart failure, O_2_ saturation at admission, chronic kidney disease, chronic liver disease, dementia, cancer, use of angiotensin-converting enzyme inhibitor (ACEi) or angiotensin II receptor antagonists (ARBs), ratio neutrophil/lymphocytes, blood urea nitrogen, use of systemic corticosteroids during hospitalization, and CURB-65 ≥ 3 and rate or ARDS moderate or severe as a markers of severity of the disease at admission). A multivariable logistic regression model also including antimicrobial, immunomodulatory, and anticoagulant therapies used during the hospitalization period of patients were calculated. Analyses were completed in IBM^®^ SPSS^®^ Statistics for Macintosh version 20 (Release 20.0.0, Armonk, NY, USA). We considered statistically significant results *p*-values < 0.05, and all tests were one-sided.

## 3. Results

A total of 537 patients with COVID-19 were admitted in five hospitals in the regions of Córdoba, Málaga, and Jaén in the south of Spain between 14 February and 5 May 2020 and were included in the analysis. Of these patients, 79 (14.7%) received calcifediol treatment, and 458 (85.3%) did not (Figure 1).

The characteristics of patients at admission according to the group of treatment are shown in Table 1. The non-calcifediol-treated group included more patients with chronic kidney disease and without significant differences in age, gender, smoking status, diabetes, hypertension, cerebrovascular disease, chronic obstructive lung disease, cancer, heart failure, coronary artery disease, or dementia. However, the supplemented groups had a higher ratio of any comorbidity than the non-supplemented group. Non-supplemented patients had lower rate of O_2_ saturation at admission (93 ± 6 vs. 95 ± 4), higher levels of CRP, higher blood urea nitrogen, and higher rates of CURB65 ≥ 3 and ARDS (moderate or severe) at admission without significative differences in neutrophil-to-lymphocyte ratio, LDH, D-dimer, ferritin, or rate of systemic corticosteroids use. The rate of ARDS (moderate or severe) at baseline in the untreated groups was 25% vs. 10% in patients that were treated with calcifediol, and no statistical differences were found in the rate of orotracheal intubation during hospitalization (6 vs. 4, *p* = 0.4). Only four patients developed disseminated intravascular coagulation (DIC), with all of them in the untreated group. Other medications dispensed are summarized in Table S1.

Baseline characteristics of patients according to center of recruitment are presented in Supplementary Table S2.

### Primary Outcome

**Figure 2:**
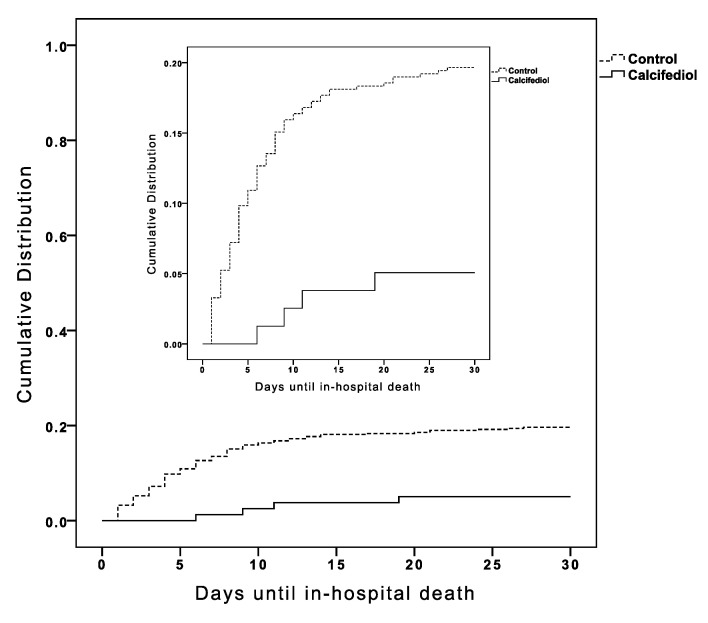
Cumulative distribution of patients presenting in-hospital death according to treatment groups.

Overall, in-hospital mortality during the first 30 days was 17.5%. Cumulative distribution of in-hospital death according to treatment group is shown in Figure 2. Patients supplemented with calcifediol had lower risk of death during hospitalization (5% vs. 20%, *p*-value < 0.01).

The OR of death for patients receiving calcifediol was 0.22 (95% CI, 0.08–0.61), *p*-value < 0.01, compared to patients not receiving supplementation.

In multivariate analysis, including potential confounders into the logistic regression model, calcifediol treatment remained statistically significant (OR = 0.16, 95%CI = 0.03–0.80) with age, center, CURB-65 ≥ 3, ARDS (moderate or severe) at admission, neutrophil/lymphocytes ratio, prior history of cerebrovascular disease, chronic obstructive pulmonary disease, and cancer as other independent predictors of mortality (Table 2).

The multivariable logistic model developed also included in analyses antimicrobial, immunomodulatory, and anticoagulant therapies used during the hospitalization period of patients, and OR of death for patients treated with calcifediol was 0.2 (95% CI, 0.04–0.9, *p*-value = 0.04) compared to patients not receiving that treatment.

The number of patients who died among the 53 control patients in center A and their distribution of confounders versus patients treated with calcifediol are showed in Supplementary Table S3. The statistically significant variables of multivariable logistic regression model for risk of in-hospital death in Center A were calcifediol treatment (OR = 0.01, 95%CI = 0.001–0.5) and ratio NL (OR = 1.6, 95%CI = 1.1–2.3) (Supplementary Table S4).

Even after selecting those patients over 65 years of age and with oxygen saturation levels at admission <96%, treatment with calcifediol was associated with a lower risk of mortality (OR 0.06, 95%CI = 0.04–0.8) (Supplementary Table S5).

## 4. Discussion

In this multicenter observational study of 537 COVID-19 patients, those who received calcifediol had a lower mortality rate during the first 30 days of hospitalization compared to patients not receiving this treatment.

This was a retrospective, observational, non-randomized study, and therefore it is no surprise that the two groups had different baseline characteristics. The treated group had a higher overall risk of comorbidity, whereas the non-supplemented group had lower values of O_2_ saturation at admission, higher CURB-65 score and higher rate of ARDS moderate or severe, higher levels of inflammatory markers such as CRP, and a higher frequency of kidney failure. Also, in the center where the treatment under study was administered, untreated patients were older, had worse prognostic markers, a higher percentage of comorbidities, dementia, or severity at the time of admission compared to those who received treatment under clinical criteria. However, the protective effect of calcifediol remained significant after adjustment for multiple confounder factors related to severity disease even after selecting those subjects who were older (≥65 years) and had worse oxygen saturation levels at admission (<96%).

Previously, our group reported in a pilot study of 76 consecutive patients hospitalized with COVID-19 infection that treatment with calcifediol decreased the need for ICU treatment or respiratory assistance [20]. In the present study, we report a lower in-hospital mortality rate during the first 30 days of hospitalization without statistical differences in the use of orotracheal intubation. This last fact could be related to the lack of normalized criteria for ICU admission between hospitals.

In terms of mechanisms, the modulatory ability of the vitamin D endocrine system in host responses to SARS-CoV-2 has been previously identified in the literature, in both the viremic and hyperinflammatory stages of COVID-19 infection [15,16,17]. Based on many models, there is a clear argument that the VDES/VDR-signaling system can defend against ARDS/acute lung damage by reducing cytokine and chemokine storm and protecting the integrity of the pulmonary epithelial barrier [26,27,28,29,30]. Cells of the immune system and cuboidal-alveolar epithelial coating cells type II (AECII) have the potential to produce 1,25(OH)2D3 or calcitriol from calcifediol (25OHD3) [13]. Thus, this synthesized calcitriol has the ability to modulate the expression of genes involved in the innate immune response, resulting in the production of antimicrobial peptides (such as cathelicidin), defensins (beta-defensine-2), and other components involved in pathogen intracellular destruction (such as toll-like receptors co-receptor CD14) [31]. Furthermore, dsRNA increases the regulation of 1-hydroxylase and sequentially synergizes with calcifediol and calcitriol to induce cathelicidin in viral infection models [31]. Calcifediol and calcitriol were equipotent in the in vivo animal model and (in vitro) on AECII cells [28,31] and the immune system [32,33], suggesting that AECII cells and activated macrophages and lymphocytes were able to actively convert calcifediol to calcitriol.

Vitamin D (25OHD) deficiency had been associated with severity and mortality of patients with ARDS from various causes [34,35,36] and in SARS-CoV-2 infected patients. Observational studies provide evidence that serum concentrations of 25OHD are inversely correlated with the incidence or severity of COVID-19. Thus, ecological studies have reported inverse correlations between historical mean concentrations of 25OHD levels and the incidence and mortality of COVID-19 in European countries [19,37]. Lower concentrations of circulating 25OHD have also been reported to be associated with an increased COVID-19 risk [38,39,40,41] and even with COVID-19 progression and severity [42,43,44,45]. Of course, acute illness or inflammation may decrease serum concentrations of 25 OHD, thereby influencing the interpretation of such observational data [46].

A recent meta-analysis of observational studies evaluating vitamin D levels in adult and elderly subjects with COVID-19 found a pattern for a link between 25OHD deficiency and COVID-19 health outcomes [47].

Additionally, the risk of severe COVID-19 infection and a 25OHD deficit overlaps with other conditions such as aging, Black or Asian ethnicity, obesity, and poverty [48]. Serum 25OHD is frequently decreased during acute inflammatory diseases [49], and 25OHD is thought to be a negative acute-phase reactant. Furthermore, to correct 25OHD deficiency in seriously ill patients [50] or COVID-19 [51], higher than usual doses of cholecalciferol are needed. For this reason, we used calcifediol as a treatment in hospitalized, severe COVID patients. Calcifediol may have some advantages over cholecalciferol [52,53]. It has a near-100 percent intestinal absorption rate and can quickly restore serum 25OHD concentrations since it does not require hepatic 25-hydroxylation. This fact is crucial in clinical circumstances where rapid restoration of serum 25OHD is desired but CYP2R1 expression is hampered, as it may be with COVID. CYP2R1 activity has been shown to be impaired in several animal models [54] and has also been confirmed in COPD and asthma patients [55].

The use of vitamin D3 in boluses administered during hospitalization for COVID-19 has not been shown to reduce the risk of death in studies carried out in Brazil (200,000 IU/5000 µg) [56] or in France (80,000 IU/2000 µg) [57]. However, the results of forthcoming, well-designed RCTs will provide new and additional information on the relevance of vitamin D in COVID-19 [58]. In a small, randomized, placebo-controlled trial from India in 25-hydroxyvitamin D deficient (<20 ng/mL) COVID-19 patients, 62.5% of participants treated with 60,000 IU/1500 µg/day of vitamin D3 for 7–14 days became negative for SARS-CoV-2 within 21 days compared to only 20.8% in the untreated participants [51]. Moreover, regular supplementation with vitamin D3, at least in the elderly, in boluses administered regularly during the year prior to diagnosis has shown a reduction in the risk of death and clinical improvement in elderly patients with COVID-19 [59,60,61].

Unfortunately, serum levels of 25OHD were not available at baseline or during treatment for the exceptional conditions of the first outbreak of COVID-19 [62,63]. According to previous reports, in late winter and early spring, adults in the Córdoba area are relatively 25OHD deficient [64,65]. It is worth mentioning that deficiency (25OHD < 20 ng/mL) in COVID-19 patients recently admitted to our hospitals has been repeatedly confirmed (data not shown here); in addition, in subsequent studies at the Reina Sofia University Hospital of Córdoba, we verified increase of serum 25OHD above 30–40 ng/mL from the third day of treatment in COVID-19 patients with the proposed calcifediol dosages.

The facts in the literature and the data in this manuscript appear to support principles related to causality, such as strength of association, consistency, temporality, biological gradient, plausibility, and coherence [66]. Our pilot study was the first experimental verification trial reported [20] that additionally encouraged the present study. These two studies together seem to indicate that Calcifediol treatment can reduce the severity of the disease.

The strengths of this study include a cohort of patients hospitalized for COVID-19 in five hospitals of different levels. However, this study has several limitations. First, this is an observational study conducted on patients admitted during the first wave of patients suffering COVID-19. Given that the administration of the treatment was not randomized, and it was based on the clinical judgment of the professionals who attended these patients, the adjustments described above have been made to try to control the possible effect on final mortality of variables that showed statistical significance at the time of admission. However, possible non-identifiable remaining confounding is possible. Secondly, since the data were obtained from the databases of the electronic medical records, we were only able to include those collected in these records for the present study. Data, such as the date of symptom onset before admission, the patients’ body mass index, the presence of other comorbidities, and the rate of development of acute kidney injury, among others, were missing in most patients and could not be included. Finally, the post-discharge follow-up events have not been taken into consideration due to lack of such information.

## 5. Conclusions

In conclusion, in this multicenter observational study, patients hospitalized with COVID-19 and treated with calcifediol had lower in-hospital mortality during the first 30 days compared to those patients not supplemented. The observational design and sample size may limit the interpretation of these findings. Results from large-scale, randomized, controlled trials of calcifediol, which are currently underway, are required for validation of our observations.

## Figures and Tables

**Table 1:** Baseline characteristics of patients treated or not with calcifediol, days of hospitalization, and mortality after 30 days of follow-up.

	Not Treated (*n* = 458)	Treated (*n* = 79)	*p*-Value
Age	67 ± 16	69 ± 15	0.23
Male (%)	60	53	0.15
Current smokers (%) ^e^	5	3	0.26
CURB-65 ≥ 3 (%)	21	8	<0.01
ARDS moderate or severe (%)	25	10	<0.01
Any comorbidity (%)	68	87	<0.01
Diabetes (%)	20	20	0.5
Hypertension (%)	56	58	0.4
Cerebrovascular disease (%)	7	6	0.53
COPD (%)	8	3	0.06
Heart failure (%)	9	5	0.16
Chronic kidney disease (%)	8	1	0.02
Cancer (%)	5	4	0.23
Coronary heart disease (%)	12	9	0.29
Dementia (%)	8	8	0.60
ACEi/ARBs (%)	48	39	0.13
SaO2 at admission	93 ± 6	95 ± 4	0.03
CRP ^a^	130 ± 100	100 ± 80	0.04
Lymphocytes	1150 ± 820	970 ± 480	0.05
Neutrophil-to-lymphocyte ratio	7 ± 7	6 ± 5	0.66
LDH ^b^	340 ± 170	330 ± 150	0.50
D-dimer ^c^	2500 ± 7200	1900 ± 5000	0.48
Ferritin ^d^	950 ± 1210	650 ± 680	0.07
Blood urea nitrogen	22 ± 19	16 ± 15	0.01
Systemic corticosteroids (%)	45	38	0.15
Orotracheal intubation, *n* (%)	26 (6)	3 (4)	0.36
Mortality, *n* (%)	90 (20)	4 (5)	<0.001

^a.^ CRP *n* = 346; ^b.^ LDH *n* = 480; ^c.^ D-dimer *n* = 480; ^d.^ Ferritin *n* = 296; ^e.^ Smoking status *n* = 508. Results are mean ± SD or % as indicated. *p*-values were calculated with Fisher’s exact test for categorical variables (Exact Sig. 1-sided) and the Student’s t-test or Kruskal–Wallis test for continuous ones. Abbreviations: ACEi/ARBs, angiotensin-converting enzyme inhibitor or angiotensin II receptor antagonists; ARDS, acute respiratory distress syndrome; COPD, chronic obstructive pulmonary disease; CRP, c-reactive protein; CURB-65, CURB-65 score for pneumonia severity; LDH, lactate dehydrogenase; SaO2, arterial oxygen saturation.

**Table 2:** Statistically significant variables of multivariable logistic regression model for risk of in-hospital death.

	OR	95%CI	*p*-Value
Calcifediol treatment	0.16	0.03–0.80	0.02
Age	1.05	1.01–1.09	0.008
ARDS (moderate or severe)	44	17–115	<0.001
CURB-65 ≥ 3	2.8	1.20–6.7	0.01
Cerebrovascular disease	3.5	1.03–11.6	0.045
COPD	9.2	2.5–34	0.01
Cancer	5.2	1.81–15	0.002
Ratio N/L	1.06	1.00–1.12	0.047
Center			
Center A	1 (Ref.)	1 (Ref.)	
Center B	0.25	0.06–1.01	0.052
Center C	0.26	0.06–1.10	0.07
Center D	0.70	0.01–0.46	0.006
Center E	0.31	0.09–0.99	0.048

Odd ratios (OR) and 95% confidence intervals (95%CI) have been calculated with multivariable logistic regression adjusted for date of hospitalization before or after approval of calcifediol treatment in Center A, age, gender, center, diabetes, chronic lung disease, smoking status, hypertension, coronary artery disease, cerebrovascular disease, congestive heart failure, O_2_ saturation at admission, chronic kidney disease, chronic liver disease, dementia, cancer, use of angiotensin-converting enzyme inhibitor (ACEi) or angiotensin II receptor antagonists (ARBs), ratio neutrophil/lymphocytes, blood urea nitrogen, use of systemic corticosteroids during hospitalization, CURB-65 ≥ 3, ARDS moderate or severe, and use of calcifediol. Abbreviations: ARDS, acute respiratory distress syndrome; COPD, chronic obstructive lung disease; CURB-65, CURB-65 score for pneumonia severity; Ratio N/L, ratio neutrophil/lymphocytes; Center A, Hospital Universitario Reina Sofia (Córdoba, Spain); Center B, Hospital Costa del Sol (Marbella, Spain); Center C, Hospital Alto Gualdalquivir (Andújar, Spain); Center D, Hospital Montilla (Córdoba, Spain); Center E, Hospital Universitario Regional (Málaga, Spain).

## Data Availability

Some or all datasets generated during and/or analyzed during the current study are not publicly available but are available from the corresponding author on reasonable request.

## Supplementary Materials

The following are available online at https://www.mdpi.com/article/10.3390/nu13061760/s1: Supplementary Table S1: Antimicrobial, immunomodulatory, and anticoagulant therapies used during the hospitalization period of patients included in analysis according to treatment (*n* = 537); Supplementary Table S2: Baseline characteristics of patients according to center; Supplementary Table S3: Baseline characteristics of patients treated or not with calcifediol, days of hospitalization, and mortality after 30 days of follow-up in Center A; Supplementary Table S4: Statistically significant variables of multivariable logistic regression model for risk of in-hospital death in Center A; Supplementary Table S5: Sensitivity analysis with statistically significant variables of multivariable logistic regression model for risk of In-Hospital Death in selected patients (>65 years and O2 saturation < 96%).
