# Supplementary material for: Calcifediol Treatment and Hospital Mortality Due to COVID-19: A Cohort Study
Source: Nutrients. 2021 May 21;13(6):1760. doi: 10.3390/nu13061760 (PMC8224356; doi:10.3390/nu13061760)
Supplement: Supplementary file 1 [file nutrients-13-01760-s001.zip › nutrients-1211439-supplementary.pdf]

Supplemental Table 1. Antimicrobial, immunomodulatory and anticoagulant therapies used during the hospitalization period of patients included in analysis according to treatment (n=537).

	Total N(%) (n= 537)	Not-Treated N (%) (n= 458)	Treated N (%) (n= 79)	p-value
**Antimicrobial therapy**				
**Beta-lactam antibiotics**	352 (66)	275 (60)	77 (98)	<0.01
**Quinolone antibiotics**	107 (20)	101 (22)	6 (8)	<0.01
**Hydroxychloroquine**	461 (86)	385 (84)	76 (96)	<0.01
**Azithromycin**	406 (76)	327 (71)	79 (100)	<0.01
**Lopinavir/ritonavir**	184 (34)	172 (38)	12 (15)	<0.01
**Immunomodulatory therapy**				
**Systemic corticosteroids**	236 (44)	206 (45)	30 (38)	0.2
**Interferon beta-1B**	54 (10)	48 (11)	6 (8)	0.3
**Tocilizumab**	58 (11)	51 (11)	7 (9)	0.4
**Bevacizumab**	4 (1)	0 (0)	4 (5)	<0.01
**Colchicine**	3 (1)	3 (1)	0 (0)	0.6
**Anakinra**	3 (1)	3 (1)	0 (0)	0.6
**Immunoglobulin**	5 (1)	0 (0)	5 (6)	<0.01
**Anticoagulant therapy**				
**Oral anticoagulants**	13 (2)	13 (3)	0 (0)	0.3
**Low-molecular-weight heparin**				
**No**	86 (16)	83 (18)	3 (4)	<0.05
**Low(prophylaxis dose)**	344 (64)	297 (65)	47 (60)	NS
**Intermediate dose**	49 (9)	36 (8)	13 (17)	<0.05
**High (anticoagulant) dose**	58 (11)	42 (9)	16 (20)	<0.05

Results are n (%). P values were calculated with Fisher's exact test for categorical variables (Exact Sig. 1-sided).

Supplemental Table 2. Baseline characteristics of patients according to center.

	Center A (n=132)	Center B (n=89)	Center C (n=62)	Center D (n=34)	Center E (n=220)
**Age**	72.5 ± 13.7	63.3 ± 16.9	65.5 ± 17.9	74.3 ± 13.5	64.8 ± 15.6
**Male (%)**	49.2	66.3	64.5	61.8	60.0
**CURB-65 ≥ 3 (%)**	18	14	31	21	19
**ARDS moderate or severe (%)**	17	26	27	21	25
**Current-smokers (%)**	2.3	7.9	3.2	2.9	5.5
**Any comorbidity**	90.2	48.3	67.7	73.5	69.5
**Diabetes (%)**	20.5	13.5	29.0	20.6	20.0
**Hypertension (%)**	66.7	37.1	59.7	58.8	55.5
**Cerebrovascular disease (%)**	8.3	3.4	3.2	17.6	6.8
**COPD (%)**	5.3	4.5	6.5	5.9	9.5
**Heart failure (%)**	4.5	5.6	8.1	5.9	12.7
**Chronic kidney disease (%)**	2.3	2.2	8.1	8.8	10.5
**Chronic liver disease (%)**	3.8	1.1	4.8	2.9	6.4
**Cancer (%)**	9.8	4.5	8.1	14.7	15.5
**Coronary heart disease (%)**	7.6	7.9	1.6	0.0	10.5
**Dementia (%)**	12.9	3.4	14.5	8.8	8.6
**ACEi/ARBs (%)**	53.0	32.6	56.5	47.1	43.0
**SaO2 at admission**	93.6 ± 5.3	92.2 ± 8.5	92.6 ± 5.2	93.2 ± 5.1	93.6 ± 4.5
**CRP[a]**	100.4 ± 81.5	114.1 ± 97.9	50.3 ± 45.4	136.5 ± 94.5	143.0 ± 101.8
**Lymphocytes**	1055 ± 532	1089 ± 511	1104 ± 624	1560 ± 2095	1117 ± 644
**Neutrophil-to-lymphocyte ratio**	6.7 ± 5.5	7.3 ± 7.3	5.8 ± 4.4	7.4 ± 6.4	6.7 ± 7.8
**LDH[b]**	341 ± 171	377 ± 225	352 ± 141	288 ± 72	325 ± 149
**D-dimer[c]**	3304 ± 10837	2050 ± 3679	2475 ± 6052	2604 ± 6212	1922 ± 4341
**Ferritin[d]**	706 ± 799	1148 ± 1251	887 ± 1257	924 ± 1090	953 ± 1269
**Blood Urea Nitrogen**	20 ± 16	20 ± 18	24 ± 16	28 ± 30	21 ± 20
**Systemic Corticosteroids (%)**	29.5	41.6	64.5	64.7	44.5
**Orotracheal Intubation, n(%)**	4(3)	11(12.4)	6(9.7)	2(5.9)	6(2.7)
**Mortality , n (%)**	24(18.2)	12(13.5)	11(17.7)	4(11.8)	43(19.5)

Results are mean ± SD or % as indicated. Abbreviations: ACEi/ARBs: angiotensin-converting enzyme inhibitor or angiotensin II receptor antagonists; ARDS: Acute respiratory distress syndrome; COPD: Chronic obstructive pulmonary disease; CRP, c-reactive protein; CURB-65, CURB-65 Score for Pneumonia Severity; LDH: Lactate dehydrogenase; SaO2: arterial oxygen saturation; Center A: Hospital Universitario Reina Sofia (Cordoba, Spain); Center B: Hospital Costa del Sol (Marbella); Center C: Hospital Alto Gualdalquivir (Andujar); Center D: Hospital Montilla (Cordoba, Spain); Center E: Hospital Universitario Regional (Malaga, Spain).

Supplemental Table 3. Baseline characteristics of patients treated or not with calcifediol, days of hospitalization and mortality after 30 days of follow-up in Center A.

	Not treated (n=53)	Treated (n=79)	p-value
**Age**	77 ± 11	69 ± 15	<0.01
**Male (%)**	43	53	0.2
**CURB-65 ≥ 3 (%)**	34	7	<0.01
**ARDS moderate or severe (%)**	26	10	0.01
**Current-smokers (%)**	1.9	2.5	0.7
**Any comorbidity (%)**	94	87	0.2
**Diabetes (%)**	20	20	0.6
**Hypertension (%)**	79	58	0.01
**Cerebrovascular disease (%)**	11	6	0.2
**COPD (%)**	9	3	0.1
**Heart failure (%)**	4	5	0.5
**Chronic kidney disease (%)**	4	1	0.4
**Cancer (%)**	11	9	0.4
**Coronary heart disease (%)**	8	8	0.6
**Dementia (%)**	21	8	0.03
**ACEi/ARBs (%)**	74	39	<0.01
**SaO2 at admission**	92 ± 7	95 ± 4	0.02
**CRP**[a]	102 ± 86	100 ± 79	0.9
**Lymphocytes**	1188 ± 587	968 ± 476.3	0.02
**Neutrophil-to-lymphocyte ratio**	7 ± 6	6 ± 5	0.4
**LDH**[b]	364 ± 195	327 ± 154	0.2
**D-dimer**[c]	5636 ± 16371	1916 ± 4991	0.1
**Ferritin**[d]	839 ± 1056	654 ± 678	0.4
**Blood Urea Nitrogen (mg/dL)**	25 ± 16	16 ± 16	0.01
**Systemic Corticosteroids (%)**	17	38	0.01
**Orotracheal Intubation, n(%)**	1(1.9)	3(3.8)	0.5
**Mortality , n (%)**	20(38)	4 (5)	<0.01

[a]CRP n=126 ; [b]LDH n= 122 ; [c]D-dimer: 126; [d]Ferritin n=82.

Results are mean ± SD or % as indicated. P value were calculated with Fisher's exact test for categorical variables (Exact Sig. 1-sided) and the Student's t-test or or Kruskal-Wallis test for continuous ones. Abbreviations: ACEi/ARBs: angiotensin-converting enzyme inhibitor or angiotensin II receptor antagonists; ARDS, Acute respiratory distress syndrome; COPD: Chronic obstructive pulmonary disease; CRP, c-reactive protein; CURB-65, CURB-65 Score for Pneumonia Severity; LDH: Lactate dehydrogenase; SaO2: arterial oxygen saturation.

Supplemental Table 4. Statistically significant variables of multivariable logistic regression model for risk of In-Hospital Death in Center A.

	OR	95%CI	P value
**Calcifediol treatment**	0.01	0.001 - 0.5	0.02
**Ratio N/L**	1.6	1.1 – 2.3	0.01

Odd ratios (OR) and 95% confidence intervals (95%CI) have been calculated with multivariable logistic regression adjusted for date of hospitalization before or after approval of Calcifediol treatment in Center A, age, gender, diabetes, chronic lung disease, smoking status, hypertension, coronary artery disease, cerebrovascular disease, congestive heart failure, O2 saturation at admission, chronic kidney disease, chronic liver disease, dementia, cancer, use of angiotensin-converting enzyme inhibitor or angiotensin II receptor antagonists, ratio neutrophil/lymphocytes, blood urea nitrogen, use of systemic corticosteroids during hospitalization, CURB-65 ≥3, and use of calcifediol.)

Abbreviations: COPD: chronic obstructive lung disease; CKD: chronic kidney disease; CURB-65: CURB-65 Score for Pneumonia Severity; Ratio N/L: ratio neutrophil/lymphocytes.

Supplemental Table 5. Sensitivity analysis with statistically significant variables of multivariable logistic regression model for risk of In-Hospital Death in selected patients (≥65 years and O2 saturation < 96%).

	OR	95%CI	P value
**Calcifediol treatment**	0.06	0.04 - 0.8	0.04
**Age**	1.2	1.1 - 1.3	<0.01
**O2 saturation at admission**	0.9	0.8-0.9	<0.01
**CURB-65 ≥ 3**	2.6	1.3-5.1	0.01
**Cancer**	4.9	1.3 – 18	0.02

Odd ratios (OR) and 95% confidence intervals (95%CI) have been calculated only in patients ≥65 years and O2 saturation < 96% with multivariable logistic regression adjusted for date of hospitalization before or after approval of Calcifediol treatment in Center A, age, gender, center, diabetes, chronic lung disease, smoking status, hypertension, coronary artery disease, cerebrovascular disease, congestive heart failure, O2 saturation at admission, chronic kidney disease, chronic liver disease, dementia, cancer, use of angiotensin-converting enzyme inhibitor or angiotensin II receptor antagonists, ratio neutrophil/lymphocytes, blood urea nitrogen, use of systemic corticosteroids during hospitalization, CURB-65≥3, and use of calcifediol.)
Abbreviations: COPD: chronic obstructive lung disease; CURB-65: CURB-65 Score for Pneumonia Severity.
